# Electricity Theft Detection in Smart Grids Using a Hybrid BiGRU–BiLSTM Model with Feature Engineering-Based Preprocessing

**DOI:** 10.3390/s22207818

**Published:** 2022-10-14

**Authors:** Shoaib Munawar, Nadeem Javaid, Zeshan Aslam Khan, Naveed Ishtiaq Chaudhary, Muhammad Asif Zahoor Raja, Ahmad H. Milyani, Abdullah Ahmed Azhari

**Affiliations:** 1 Department of Electrical and Computer Engineering, International Islamic University, Islamabad 44000, Pakistan; 2 Department of Computer Science, COMSATS University Islamabad, Islamabad 44000, Pakistan; 3 Future Technology Research Center, National Yunlin University of Science and Technology, 123 University Road, Section 3, Douliou, Yunlin 64002, Taiwan; 4 Department of Electrical and Computer Engineering, King Abdulaziz University, Jeddah 21589, Saudi Arabia; 5 The Applied College, King Abdulaziz University, Jeddah 21589, Saudi Arabia

**Keywords:** electricity theft detection, smart grids, robustness, smart meters, Tomek links

## Abstract

In this paper, a defused decision boundary which renders misclassification issues due to the presence of cross-pairs is investigated. Cross-pairs retain cumulative attributes of both classes and misguide the classifier due to the defused data samples’ nature. To tackle the problem of the defused data, a Tomek Links technique targets the cross-pair majority class and is removed, which results in an affine-segregated decision boundary. In order to cope with a Theft Case scenario, theft data is ascertained and synthesized randomly by using six theft data variants. Theft data variants are benign class appertaining data samples which are modified and manipulated to synthesize malicious samples. Furthermore, a K-means minority oversampling technique is used to tackle the class imbalance issue. In addition, to enhance the detection of the classifier, abstract features are engineered using a stochastic feature engineering mechanism. Moreover, to carry out affine training of the model, balanced data are inputted in order to mitigate class imbalance issues. An integrated hybrid model consisting of Bi-Directional Gated Recurrent Units and Bi-Directional Long-Term Short-Term Memory classifies the consumers, efficiently. Afterwards, robustness performance of the model is verified using an attack vector which is subjected to intervene in the model’s efficiency and integrity. However, the proposed model performs efficiently on such unseen attack vectors.

## 1. Introduction

Power generation, transmission and distribution collectively build a power system infrastructure. The power generation phase generates electricity at a high voltage level. The generated electricity is supplied to the end user through transmission lines. The end user is the consumer who consumes the supplied electricity via distribution network [1]. Smart Meters (SMs) are installed on the end users’ side by Utility Providers (UPs) in order to monitor the consumed energy [2]. There are two types of losses, Technical Losses (TLs) and Non-Technical Losses (NTLs) [3]. TLs are the network-associated losses, which are confined to the design and material of the infrastructure, while NTLs are the losses which occur due to the interruption of the end consumers to obtain financial benefits by under-reporting the consumed energy. The interruption of the end consumer is basically a malicious activity, which is adopted by the fraudulent consumers. The connected fraudulent consumers tend to tamper the net metering of their consumed energy by adopting various data tampering techniques, such as meter tampering using shunt devices, double tapping of the lines and electronic faults [4]. The effects of such malicious activities over-burden the UPs with huge financial losses, which disrupt the smooth energy flow and demand curve. For instance, the study conducted in [5] reports that the monitored losses have been increased from 11 percent to 16 percent during the last two decades (1980–2000). The increased losses clearly highlight that revenue losses due to NTLs are a conspicuous issue and need special attention. NTLs vary from country to country. The literature in [6] reports that about 20% of the total revenue loss in Indian electricity network is due to the aforementioned malicious activities. Similarly, the United States is also facing a revenue loss of USD 6 billion annually [7,8]. Worldwide, revenue losses of about USD 96 billion are reported due to such malicious activities [9].

In order to investigate the aforementioned problems, the literature suggests various counter measure approaches to reduce such losses. The suggested approaches are advance metering infrastructure (AMI) and Neighborhood Area Network (NAN) [10], which are hardware-based approaches. In AMI, a sequential data is a target parameter, which is analyzed to extract suspicious behavior in order to find out maliciousness. Furthermore, consideration of sequential and non-sequential information enhances the detection of malicious behavior. Sequential data are Time-Series Data of the consumers, whereas non-sequential data are an auxiliary data that contain attributes of geographical, demographical and topographical data. Moreover, NAN and morphological patterning assessment focuses on multiuser network-based detection. A NAN is a multiple consumer network where a master meter is deployed to monitor the total consumed energy. A master meter is connected to a distribution low-voltage side of the transformer, which works as an observer meter to monitor the cluster of the connected SMs. TLs of the distribution lines are numerically adjusted as a beta σ, which is added to the total network’s consumption. The data relevancy of the network is observed in order to investigate the maliciousness. Total consumption in addition with the σ factor is related to the observer meter’s reading. Furthermore, in morphological patterning analysis, a historic and forecasted data competency is measured, which is correlated based on the error factor. A threshold is set as a monitoring parameter which analyzes the parity check of each of the consumptions and reports malicious activity.

Based on the above analysis, the motivation is to propose a data-oriented approach to detect NTLs. The problem of imbalanced data, defused decision boundary and extraction of abstract features are the main factors to target through data-oriented-based analysis of the Time-Series Data.

## 2. List of Contributions

The contributions are as follows:

- To tackle the imbalance data issue, theft class data are synthesized using six theft variants. Later on, the synthesized data are oversampled using a K-means synthetic minority oversampling technique (SMOTE).
- A Tomek links technique is used to eliminate cross-pairs across the decision boundary.
- To overcome the data leakage problem, a simple stratified approach is opted for.
- Cumulative and distinct features are engineered using stochastic feature engineering, which enables the model to learn data characterization and uniqueness.
- An integrated hybrid model of Bi-Directional Gated Recurrent Units (Bi-GRU) and bi-directional long-term short-term memory (Bi-LSTM) is used to tackle misclassification and high FPR issues.
- Furthermore, to verify the robustness of the proposed model, an unseen variant of the theft data with temperate randomness is analyzed to acknowledge the stability and integrity.

## 3. Literature Review

This section overviews Electricity Theft Detection (ETD)-related proposed research activities of various authors in smart metering applications.

### 3.1. Considering Sequential Data

A major portion of NTLs is due to fraudulent behavior of the consumers’ accomplishing an effort to bypass the Utility Provider (UP) surveillance and to under-report the consumed energy. A solution proposed in [11] adopts a data-driven approach which uses a Machine Learning technique, Ensemble Bagged Tree (EBT) algorithm by stacking many Decision Trees to detect NTLs. As time complexity and memory consumption due to large computational complexity have remained formal constrains for Machine Learning (ML) algorithms. To improve both, searching and Weighted Feature Importance (WFI) techniques are deployed to enhance theft detection schematics. A Gradient Boosting Classifier (GBCs)-based detector is used to detect anomalies by considering intentional remedies while non-fraudulent anomaly intervention is ignored. Furthermore, the Gradient Boosting Theft Detector (GBTD) for the classification purposes is pursued by a preprocessing module using WFI. WFI uses stochastic features such as mean, min, max and Standard Deviation in collaboration with the consumption pattern extracted features, which improves performance and reduces time complexity [12]. The author pinpoints the Detection Rate and FPR only, however, a clustering mechanism is required to be considered in order to identify the misclassification due to a sudden drop in the consumption, which is ultimately started before the period of analysis. During training of the model, a problem of data leakage occurs which is not tackled properly. In [13], a maximal overlapped discrete wavelet packet transform is used to extract the abstract features from the dense time-series electricity consumption data, whereas, to tackle the data balancing issue, a random under-sampling boosting (RUSBoost) algorithm is proposed, which eliminates vital information of the data while re-sampling the data samples. Similarly, [14] uses SMOTE for data balancing. The balanced data are then preprocessed using a min–max scalar normalization method to refine the input raw data. A pool of various algorithms is used containing AdaBoost, Cat-Boost, XGBoost, LGBoost, RF [15] and extra trees to find FPR and Detection Rate, however, SMOTE over-samples the minority class, with confused pairs having trace contents of both classes. The generalization performance of single hidden-layer feed-forward neural networks (SLFN) due to over-training leads to degradation when the back-propagation algorithm performs. To overcome such issues, a hybrid Convolutional Neural Network and Fandom Forest (CNN–RF) is proposed, where the CNN is designed to learn features between different hours of the day [15]. Obtained features are taken as an input by Random Forest (RF) to segregate thieves from honest customers. However, memory elapsing is a serious issue to monitor consumption patterns for long periods of time. The RF module takes a lot of memory, causing over-fitting issues. Significantly, a fast operation is an optimum choice, whereas operating maxpooling is a slower operation and causes greater time of execution. Furthermore, due to the non-availability of real-world theft scenarios, data analyzing classification based only on linear Theft Cases is not a significant investigation scenario. Similarly, a hybrid module integrating Convolutional Neural Network and long-term short-term memory (CNN–LSTM) has been developed [4]. CNNs have the capability of self-learning, whereas LSTM performs better on sequential data, however, memory elapse is still a question for such scenarios. A Semi-Supervised Auto-Encoder (SSEA) is used to learn the advanced features [16]. The input of multiple Time-Series Data is organized as a 1D vector in multiple channels. Moreover, to improve a linear separability of the samples, a distributed stochastic neighbor embedding (t-SNE) is used to localize each data point. Adding a high dimensionality though class separation is a pre-requisite for such a scenario, which is not simply tackled by t-SNE to add dimensionality for the class separation. Data leakage during training of the model and the consideration of non-malicious factors are important aspects, however, [17] pays no attention to these issues. Furthermore, the authors in [18,19] adopt a data-driven approach using a Machine Learning technique, XGBoost, without considering any auxiliary information. The study in [20,21] investigates the impact of imbalanced data. The imbalanced data are balanced through synthesized data. The data reductionality is carried out through Principle Component Analysis (PCA) and hyper parameters are tuned through Bayesian optimizer. An AUC score of 97% is reported using a feed-forward network. The study in [22] uses a hybrid model of graph convolutional network and EU Convolutional Neural Network. CNN is used to capture the latest features. The study in [23] targets the AMI infrastructure to investigate malicious consumers. The benign data are manipulated through cyber attacks. A deep neural network CNNGRU hybrid model is developed to correlate the malicious and benign samples.

### 3.2. Monitoring Morphological Patterning

An LSTM model is used by [24,25] to investigate pattern morphology. The pattern authentication is investigated by mapping them together. A prediction error is calculated between the real and predicted consumption, which decides the authenticity of the consumed pattern. However, due to excessive computational complexities, LSTM is not a suitable option. The authors in [26] propose a Stacked Sparse Denoising Auto-Encoder (SSDAE), which monitors the reconstruction error of the corresponding consumption pattern based on the extracted features. The extracted key features from the raw samples are provided as an input. A comparative correlation is observed between the samples provided as an input and reconstructed patterns. The similarity index is observed through an Optimized Estimated Threshold (OET). OET decides the sample’s class based on the measured value of reconstruction error (RE). However, based on non-sequential attributes, consideration of exogenous variables affects the morphology of consumers’ patterns [27]. In addition to short-term vacations, demographical, geographical, SM firmware and EM distort the pattern’s morphology, which is beyond the scope of detection, using SSDAE’s estimated threshold as a segregating boundary for the classes. Furthermore, the tampering of consumption patterns before installation of SM on customers’ premises remains undetected. The tampered pattern reconstruction significantly deceives the SSDAE detector, which causes misclassification. In [28], NTLs are categorically divided based on the time period, including consumers cheating during ON-Peak hours, OFF-Peak hours and malicious customers cheating constantly. The detection model becomes unstable when inconsistent attacks are injected. To monitor such inconsistent variations, categorical variables are incorporated in linear regression to develop a categorical variable linear regression detector. In [29], an Anomaly Pattern Detection Hypothesis Testing (APD-HT) investigates theft activities. A reference and a detection window are used to analyze the data streaming of SMs. The data streaming analysis is based on binomial data distribution. However, variations due to the intervention of non-malicious factors are beyond detection.

### 3.3. Tampering with Smart Meter Readings

In addition to the data-oriented approaches [30,31,32], another novel Distributed Generation (DG)-based approach of energy monitoring is proposed. A renewable DG unit consists of Photo-Voltaic (PV) modules, which are installed on consumers’ premises. Consumers generate energy according to their needs and sell back the excessive amount of energy to the UPs. A two-metering system is adopted, namely, net metering system and Feed-in Tariffs (FITs) policy. Net metering systems monitor consumed energy provided by the UP, while FITs policy monitors the excessive energy generated by a DG for selling purposes. Manipulating and tampering with injected (sold) readings of DG by malicious customers tends to falsely report over-charging. The work in [33] proposed a solution by deploying Supervisory Control and Data Acquisition (SCADA) metering points to monitor various electrical parameters.

### 3.4. Investigating Neighborhood Area Networks

Hardware-based infrastructure utilizes network-based topology to enhance detection performance. The authors pinpoint the limitations of misclassification due to manipulation of non-malicious factors and deceiving a detection detector to accept the malicious pattern as a normal one [34]. The authors suggest to deploy an SM on the transformer’s side, so that a balancing load flow scenario is overlooked, scrutinizing the discrepancies being caused by the non-malicious factors and smart attackers. A Neighborhood Area Network (NAN) proposes a master meter (MM) approach, which is installed on the distribution transformer side and monitors total supplied energy to the NAN [35]. The total supplied energy is compared with the sum of total individuals’ SM readings within the corresponding NAN, where TLs are accommodated by addition of a constant parameter. The inequality within the readings indicates a theft occurrence, while equality in the NAN means a complete benign consumption. A Correlation Analysis for Pinpointing Electricity Theft (CAPET) scheme is introduced, which measures the correlation between total utilized energy in the NAN at the low voltage level side. Inequality and deviation shows malicious activity. However, change in TLs is subjected to environmental conditions; a seasonal change abruptly affects the balanced correlation between MM and SM readings. Inequality in reading of the dispatched side and consumer premises indicates suspicious activity, which is beyond consideration. Similarly, in [36], the author develops an ensemble technique by combining the suspicious ranks obtained from the Maximum Information Coefficient (MIC) and clustering technique. The arithmetic and geometric means of these two ranks are combined using a famous rank product method which decides whether a sample is benign or malicious. The decision is based on the rank’s intensity. A high intensity indicates malicious activity. The MIC and clustering technique analyzes the correlation of NTLs and the observer meter, respectively. In order to identify unusual shapes, a degree of abnormality is calculated by clustering technique [37]. However, such correlations are void of consideration for variable TLs and non-sequential auxiliary data aspects.

## 4. Proposed System Model

**Figure 1:**
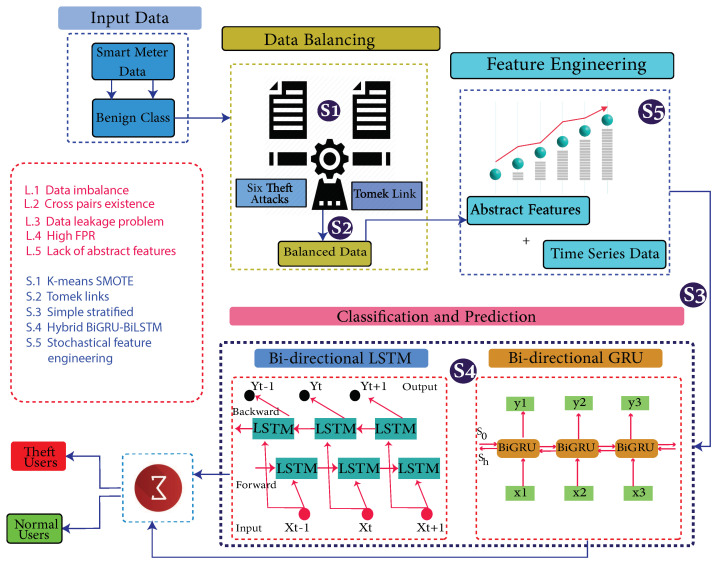
System Model Architecture.

Figure 1 shows the proposed system model, while limitations, along with their proposed solutions, are mapped in Table 1.

The system model comprises the data preprocessing module, data augmentation module and classification module. These modules are subdivided into 7 main steps.

- Step (1) is a data preprocessing step, where missing values are filled using a mean-based strategy and outliers are removed. Filling and removing such values is a necessary step of the data preprocessing, as noisy and ambiguous data affect accuracy and degrade the misclassification scenario. A simple imputer is implemented to fill such values.
- In step (2), the preprocessed data are augmented where benign samples are modified and manipulated due to their rare existence. The problems of skewness and bias are observed if the model is trained on such imbalanced data. Therefore, it is a necessary step to balance the data before the training of the model.
- In step (3), benign class data are manipulated and theft class data are generated.
- In step (4), decision boundaries’ associated cross-pairs are identified and eliminated. As cross-pair is a combination of the opposite class samples. Henceforth, a Tomek links technique is used. The majority class samples are removed, and minority class samples are retained in order to preserve the data integrity.
- In step (5), the data is stratified in order to inhibit the defusion of the data while splitting.
- In step (6), abstract features are engineered based on stochastic feature engineering.
- In step (7), Time-Series Data are inputted to a developed Bi-GRU [38] and Bi-LSTM [39]. A binary sigmoid function classifies the samples [40]. Bi-LSTM [41] is featured with the handling of high dimensional data, while Bi-GRU is used to avoid the computational complexity due to its fast operating features.

This paper is an extension of [9]. Algorithm 1 presents the BiGRU–BiLSTM-based scheme for the detection of the anomalies in smart grids. It consists of seven steps. Initially, data are segregated based on distinct characterizations. Later on, six data manipulating techniques are appertained on the honest consumers’ data, which are pursued by concatenation and data balancing techniques. Moreover, data are preprocessed and cross-pairs are removed. Furthermore, stratified sampling and feature engineering are accomplished.
**Algorithm 1:** Bi-GRU- and Bi-LSTM-based Detection Scheme.   **1** **Step 1**:   **2** Input: Benign Consumers $B_{C}$, Output: Fraudulent Consumers $F_{C}$   **3** **Step 2**: Generating Theft Samples   **4** T1 = $B_{C}$∗random(0.1,0.9);   **5** T2 = $B_{C}$$*x_{t}$ where  $(x_{t}=random\left(0.1,0.9\right));$   **6** T3 = $B_{C}$∗random[0,1];   **7** T4 = mean ($B_{C}$)∗random(0.1,1.0);   **8** T5=Mean(S)  for  each  column;   **9** T6=S(T)−t  revesing  a  time sequence; **10** **Step 3**:  concatenation **11** Concat ($B_{C}$ + $F_{C}$); **12** **Step 4**: Balancing Data **13** $B_{C}$ = $F_{C}$; **14** **Step 5**: **15** $S_{ith}$ of majority class having smaller EU Distance with decision boundary is removed; **16** **Step 6**: Data Leakage **17** p(s) = $C_{i}$  +  $C_{j}$; **18** $C_{i}$  ⊆  p(s); **19** $C_{j}$  ⊆  p(s); **20** $S_{j1},S_{j2},S_{j3},\ldots ,S_{jn}$  ε  $C_{j}$; **21** $S_{i1},S_{i2},S_{i3},\ldots ,S_{in}$  ε   $C_{i}$; **22** $S_{i}$ ∉$\;S_{j}$; **23** $C_{i}\left(S_{i1,\ldots ,n}\right)$ ∉  $C_{j}\left(S_{j1,\ldots ,n}\right)$; **24** **Step 7**: Feature Engineering **25** F1 = Mean of $P_{s}$   against  each row; **26** F2 = Std of $P_{s}$   against  each row; **27** F3 = Min∈$C_{i}$  against  each row; **28** F4 = Max∈$C_{j}$  against each row; **29** Output: Honest Consumers ε$B_{C}$, Fraudulent Consumers ε$F_{C}$.

### 4.1. Dataset

A realistic electricity consumption dataset, namely, the State Grid Corporation of China (SGCC), is used in this paper. It is administered during the 2014–2016 period and is supposed to be one of the most extensive datasets of SMs. It is structured as Time-Series Data, which are collected after every 24 h. Each consumer has a unique household ID. The consumption volume of each consumer is recorded against their household ID along with the date and time. It is a dataset of 1035 days and 42,372 consumers. We are using 1500 benign consumers’ data of six months due to the limited resources of our machine. Machine specifications are Intel(R) core (TM) M-5y10c, CPU@ 0.80 GHz 1.00 GHz, RAM 4 GB. Moreover, The simulator is Google CoLab. The meta information of the SGCC dataset is shown in Table 2.

Generally, in a power system, the electricity consumption data of end users are collected through SMs. The collected data are acquired using various sensors of the SMs. A data communication network aggregates the data at a specific central location. However, certain complications such as the malfunctioning of the sensors, failure of the SMs, errors in data transmission and storage servers generate inherent erroneous and ambiguous data. Discarding such data shrinks the size of the dataset considerably, and thus authentic analysis of the data becomes onerous.

### 4.2. Data Leakage

The population is divided into mutually exclusive subgroups using stratified sampling. It is a homogeneous division and known as strata. The purpose of using stratified sampling is to clearly classify each strata of the samples’ population. The SGCC dataset is divided into training and testing data. The training and testing samples are segregated into subgroups by opting stratified sampling in order to avoid misclassification due to extensive diversity in the data. Training and testing samples are confined to their specific operations only. Training samples are used to train the model, whereas testing samples are exploited to validate classification and prediction. In this way, data leakage of training into testing and vice versa is reduced, which results in a good generalization. The mathematical representation of the data leakage is as follows:

(1) $$p\left(s\right)=C_{i}\;+\;C_{j}$$

(2) $$C_{i}\;\subseteq \;p\left(s\right)$$

(3) $$C_{j}\;\subseteq \;p\left(s\right)$$

(4) $$S_{j1},S_{j2},S_{j3},\ldots ,S_{jn}\;\varepsilon \;C_{j}$$

(5) $$S_{i1},S_{i2},S_{i3},\ldots ,S_{in}\;\varepsilon \;C_{i}$$

(6) $$S_{i}\notin S_{j}$$

(7) $$C_{i}\left(S_{i1,\ldots ,n}\right)\notin C_{j}\left(S_{j1,\ldots ,n}\right)$$

where p, *s* and *C* represent Population of the Samples, Number of Samples and samples’ unique class, respectively, whereas i and j are the mutual binary classes.

### 4.3. Data Preprocessing

Data is preprocessed where raw data are transformed into affine usable data. As the consumption data are highly complex in nature and dimensionality, tackling such large data manually is an impractical task, which takes much time to execute. Such complex data results in high FPR and low accuracy. Missing values in raw data are filled by applying a simple imputer, where a mean-based strategy is applied for such ambiguous values.

### 4.4. Data Augmentation and Balancing

Due to the rare existence of the malicious samples, the benign class samples’ are modified and manipulated to synthesize malicious class data, which are inputted to ML and Deep Learning (DL) models. Such random data distribution causes skewness and bias problems. To tackle such issues, over-sampling techniques are used. Under-sampling techniques discard the majority class, which disrupts the important information, while oversampling techniques synthesize the duplicate samples of the minority class, which are prone to over-fitting. In our scenario, the balanced data are synthesized by six theft variants to cope with the realistic theft data. Manipulating techniques used for the synthesis of the data are as follows [42,43,44,45,46]:

(8) $$T1\left(s_{t}\right)=s_{t}*rand\left(0.1,0.9\right)$$

(9) $$T2\left(s_{t}\right)=s_{t}*x_{t}\left(x_{t}=random\left(0.1,0.9\right)\right)$$

(10) $$T3\left(s_{t}\right)=s_{t}*\left(random\left[0,1\right]\right)$$

(11) $$T4\left(s_{t}\right)=mean\left(s_{t}\right)*random\left(0.1,1.0\right)$$

(12) $$T5\left(s_{t}\right)=mean\left(s_{t}\right)$$

(13) $$T6\left(s_{t}\right)=S_{T-t}\;\left(Where\;T\;is\;consumption\;time\right)$$

**Figure 2:**
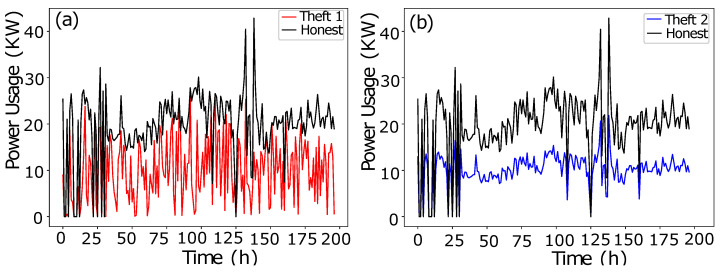
(**a**) Theft Case 1. (**b**) Theft Case 2.

**Figure 3:**
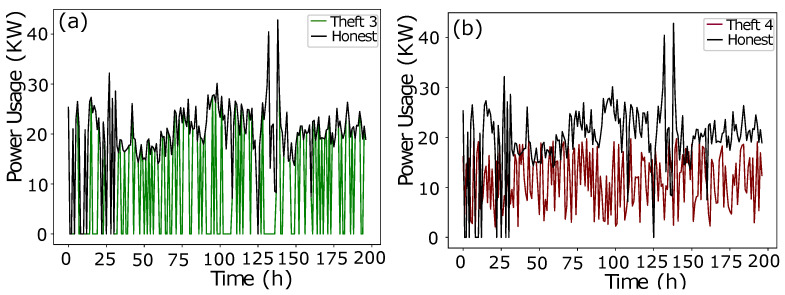
(**a**) Theft Case 3. (**b**) Theft Case 4.

**Figure 4:**
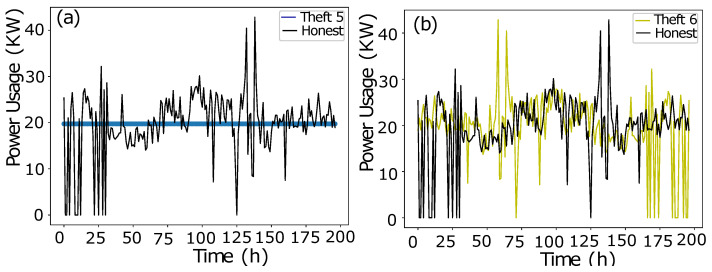
(**a**) Theft Case 5. (**b**) Theft Case 6.

- In data manipulation technique 1, as shown in Figure 2a, a random number is multiplied with benign class Time-Series Data in order to manipulate fair consumption.
- The data manipulating technique 2 is shown in Figure 2b. To capture the consumption’s discontinuity, a random number is multiplied to manipulate the honest consumption’s data. Random number multiplication is a series-based discontinuity in the consumption pattern.
- The data manipulating technique 3 is shown in Figure 3a. A random multiplication of 1 and 0 with Time-Series Data shows either the original consumption or a complete zero consumption. There is no ramping function in between 1 and 0. It is a straightforward switching ON, OFF operation with a complete connected load or the cut off. The multiplication is a mode to copy the historic consumption project, and it is not confined to a continuous Time-Series Data.
- In Theft Case 4, total consumption is aggregated into a mean which is multiplied by a random number in between (0.1, 1.0), as shown in Figure 3b.
- The data manipulating technique 5 is shown in Figure 4a. The aggregated mean is multiplied with a random number. It is a two-part manipulation. The average value is a centered value of continuous Time-Series Data, where maximum consumption is under-reported. In the second part, the same aggregated value is multiplied with a random number in between (0.1–0.9), where the average value is under-reported as well in an extra exploitation.
- The data manipulating technique 6 is shown in Figure 4b. A continuous swapping of the low consumption and peak consumption hours is practiced, where a couple slabs of consumed energy are shifted from ON-Peak hours to OFF-Peak hours and vice versa. In such manipulating techniques, the consumer pays the charges for the consumed energy, however, the vigilant swapping does not affect the UPs extensively.

### 4.5. Bi-Directional LSTM

To resolve the problem of vanishing gradients in RNNs [47], Bi-LSTM is developed to preserve information for a long time period. Bi-LSTM infrastructure consists of two LSTMs, which operate parallel in the forward and backward direction. Past and future Time-Series Data are processed through forward and backward direction gates, respectively. The input data are fed in the forward direction, and the reverse copy of the same inputted data are fed in the backward direction as well. Such nature of the inputted data with a reverse copy increases the data compatibility. The compatibility limits the gates to function accordingly as needed. The architecture contains two hidden layers, and the output layer is concatenated afterwards.

### 4.6. Feature Engineering

**Figure 5:**
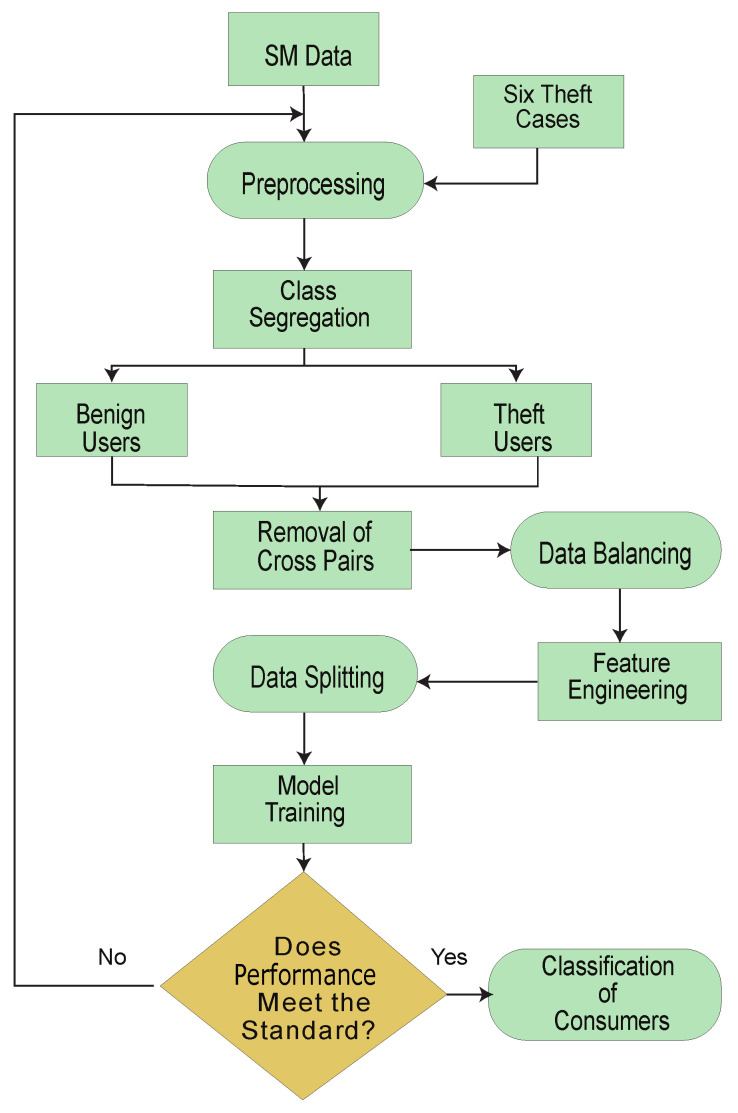
Methodology outline for detection of NTLs.

Synthetic features are helpful to improve the performance of the model. Four various types of synthetic stochastic features are generated, namely, mean, min, max and standard deviation. Time-Series Data of SGCC are analyzed on a monthly usage basis. The generation of the stochastic features creates a subset of available features, which reduces noise and improves DR slightly. However, FPR is reduced to a larger extent. The stochastic features are numeric features. Weighted Feature Importance (WFI) of these features is classifier-dependent. Certain features may not be of default importance to obtain a suitable DR and low FPR. The stochastic features are the principal important features, which contribute in our scenario. To confirm the validation, we iteratively tested and trained the classifiers on the SGCC dataset. Mathematical representation of the generated features is as follows:

(14) $$y\left(t\right)=\left\{y_{t};t=0,1,2,3,4,\ldots ,n\right\}$$

(15) $$\mu =\sum_{i}^{n}\frac{O_{n}}{T_{O}}$$

(16) $$\sigma =\sqrt{\frac{\sum_{i=0}^{n}{\left(O_{i}-\mu \right)}^{2}}{P_{y}}}$$

(17) $$Minimum=O_{sv}\left[y\left\{t_{i}\right\}\right]$$

(18) $$Maximum=O_{hv}\left[y\left\{t_{i}\right\}\right]$$

where, y(t),t,O,T,n,u,sv,hv and *P* show Time-Series Data containing various numbers of features, time spans, observations, total number of observations of a specific time sequence, number of observations, mean, smallest value, highest value and total population of the dataset, respectively. Figure 5 shows the complete flow diagram of the overall classification scenario.

## 5. Performance Evaluation

To evaluate the performance of our developed hybrid model, we use DR, FPR and AUC scores and accuracy [48]. The origin of all of the aforementioned parameters is a confusion matrix. Parametric division of the dataset is observed based on the confusion matrix in shapes of True Positive (TP), FP, True Negative (TN) and False Negative (FN). TP and TN correctly analyze the honest user as honest and malicious as malicious, respectively. FP and FN wrongly classify the samples. Similarly, a model’s detection and sensitivity are monitored by DR, which is referred to as TPR in the literature as well. Basically, DR is the representation of the model’s sensitivity and detection, which is mathematically shown in Equation (19).

(19) $$DetectionRate=\frac{TruePositive}{\left(TruePositive+FalseNegative\right)}$$

FPR is a vital evaluation factor in a detection and classification scenario to monitor the competency of a model which shows false alarms. A false alarm is an incorrect classification of positive samples as negative ones and vice versa. Such alarming parameters are quite expensive, which requires on-site inspection to verify, and it results in a huge monitory loss. To mitigate huge revenue losses, high FPR needs to be reduced. Mathematically, it is shown in Equation (20) [49].

(20) $$FPR=\frac{FalsePositive}{\left(FalsePositive+TrueNegative\right)}$$

Moreover, the accuracy is the measure of the correctly predicted instances. Mathematically, it is represented as in Equation (21).

(21) $$Accuracy=\frac{\left(TP+TN\right)}{\left(TP+TN+FP+FN\right)}$$

A suitable and good classifier is one having low FPR, high DR and high accuracy as well.

## 6. Simulation Results

The exploited data (SGCC) are a real-time residential consumer’s data. Similar indexing pattern-based morphology classifies the consumers into two classes, in perspective of their consumption, which are properly labeled. A staging numeric binary is placed for each individual consumer’s consumption pattern. Label 0 indicates a fair consumer, whereas 1 indicates a fraudulent consumer. The monitored and reordered patterns are recorded after every 24 h for each consumer. Benign class data are manipulated in order to synthesize malicious data for each of the theft variants. Later on, both classes’ data are concatenated. However, a data balancing technique is required to reduce the class bias issue due to the skewness of the model towards the majority class. K-means SMOTE is deployed to balance the data. Before provision of the data to a model for training, both classes are segregated through an affine decision boundary, where cross-pairs are removed, which degrades model detection and classification accuracy. The Tomek links technique identifies and removes the in-rushed cross-pairs across the decision boundary. The number of identified and removed samples is shown in Table 3.

**Figure 6:**
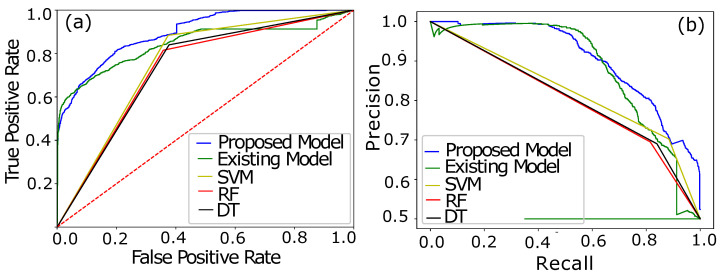
(**a**) AUC Analysis of the proposed and CNN–LSTM models. (**b**) PRC analysis of both models.

**Figure 7:**
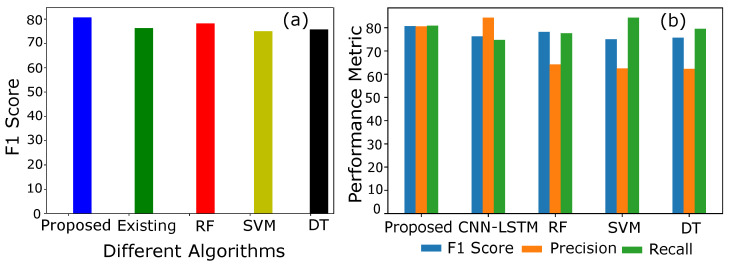
(**a**) F1 Score of different models. (**b**) Comparison of F1 Score, precision and recall.

In Figure 6a, the performance of the proposed BiGRU–BiLSTM is compared with an existing CNN–LSTM model [32]. The curves in Figure 6a indicate the AUC of the CNN–LSTM, proposed and ML-based models. Initially, at an AUC score of 0.50, both of the classifying models comparatively perform quite well, where high TPR and the lowest FPR are achieved, as shown in Figure 7a. The initial assessment based on the AUC curve shows that the CNN–LSTM model [32] classifies the samples efficiently with the recorded lowest FPR when the inputted samples passed are fewer in number. However, a small spike in the AUC curve at 0.60 shows that the data complexity moderately confuses the CNN–LSTM classification and results in an increasing FPR. The increasing FPR behavior is fluctuated in a range of AUC scores from 0.60–0.82, while during the defined ranged our proposed hybrid model Bi-GRU–Bi-LSTM performs much better to learn the data complexity and reduce FPR. The maximum AUC score of 0.93 is achieved by our proposed model with a high sensitivity rate (TPR) as compared with the opponent model. Moreover, performance of the proposed model is analyzed using a PRC curve. Figure 7b shows the performance curve of PRC, which ensures that a low PRC rate is not an optimal factor due to the high misclassification rate. Misclassification of the consumers spikes FPR and burdens the UPs due to the on-site inspection for the conformation of the consumers’ nature, which is expensive in practice due to the revenue loss.

Similarly, accuracy is not a good metric to evaluate the results of the whole classification scenario. Accuracy-based performance analysis of different models is shown in Figure 7a,b. Accuracy is the number of correct predictions over the total number of predictions. However, the prediction sometimes goes wrong and misclassifies the samples mistakenly. Figure 7b shows that CNN is a dumb classifier, and it takes advantage of the skewness of available data. To overcome the issue and to evaluate the performance of the classifier, F1 and precision scores are plotted.

The leading diagonal of the confusion matrix contains FP and FN, which are referred to as mistakes of the classifiers. A perfect classifier has the zero leading diagonal. Fluctuations in precision and recall are formally due to these two aforementioned factors.

Precision- and recall-based performance of a model is integrated into a single matrix called an F1 score. It is the harmonic mean of the precision and recall. Only a significant increase in both, i.e., precision and recall, can cause an increase in F1 score. Figure 7b shows an equilibrium in precision and recall, which results in a high F1 score, while the existing model has a low F1 score due to imbalance increase in precision and recall. Moreover, the bench mark models such as SVM, RF and DT depict the same scenario of the existing model with high fluctuations in F1 scores.

A comparative analysis in Table 4 shows a subsequent improvement in classification between the honest and fraudulent consumers. In addition, feature engineering improves the accuracy of the proposed detection model as shown in Table 5. It is observed that the accuracy is increased from 88.7% to 95%.

## 7. Robustness Analysis

Robustness shows the effectiveness of a classifier against unseen and independent samples of a similar dataset whenever it is tested on such type of data. The unseen and independent data are referred to as the worst case of noisy data due to their distinctive characterization. In our case, Theft Case 3’s data are taken to verify the robustness of the model. Theft Case 3 presents the most irregular consumption patterns as compared with the other Theft Cases due to a temperate randomness in consumption patterns, which is caused by the multiplication of the patterns with 1 and 0. The irregular and distinct patterns mimic changes as directives of inevitable factors, which proscribe the changes as suspected ones. A high-degree patterns’ variation disrupts models’ decision making. However, the proposed model survives to generalize completely on unseen data, as shown in Table 6.

Table 6 depicts the observed accuracy, AUC and F1 scores. The statistics in Table 6 show that a higher DR is achieved with a high FPR. However, the high FPR is within an acceptable range as compared with the existing model.

## 8. Computational Complexity

To analyze the computational complexity of the proposed model, execution time is considered. Table 7 shows the execution time of the proposed and existing models. It is observed that the execution time of the proposed model is slightly greater as compared with the existing model. However, our major concern is high FPR. The proposed model beats the existing model in high the FPR perspective, which is an expensive parameter. High FPR burdens the UP and results in excessive monitory costs, whereas the computational complexity is a time-oriented parameter, which can be compromised.

## 9. Performance Validation

In order to validate the effectiveness of our proposed model, a random testing on unseen theft class data is tested. The unseen theft class data are manipulated data of Theft Case 3, as shown in Equation (10). The observed AUC score of 57% validates the performance of the proposed model. Moreover, variation in the testing data due to the addition of the stochastic features challenges the performance, where an AUC score of 95% is observed. An AUC score of 95% is a good achievement and validates the performance of the proposed model.

## 10. Conclusions

This research proposes a hybrid model of BiLSTM and BiGRU in order to detect NTLs. Initially, benign and fraudulent consumers are segregated by defining an affine decision boundary through the Tomek Links techniques. Cross-pairs are identified and transformed into majority samples, where the majority class samples are removed and reduce the misclassification of the defused data across a decision boundary, which results in a low FPR. Furthermore, to synthesize theft variants, honest consumption is modified and manipulated by using six different data manipulating techniques. Six numbers of manipulated readings are synthesized for a single benign sample, which requires data balancing. For provision of the balanced benign class data, K-means SMOTE is used. K-means SMOTE over-samples the benign class using a clustering mechanism. The balanced data are inputted to the hybrid architecture of Bi-GRU–Bi-LSTM. The classification analysis is carried out on unseen data samples and achieves an AUC score of 0.93. Similarly, a competitive model of CNN–LSTM is trained and tested on the same data, which fails in the provision of a precise and accurate classification as compared with our proposed model.

## Figures and Tables

**Table 1:** Mapping of Limitations and Proposed Solutions.

Limitation Number	Limitation Identified	Solution Number	Solution Proposed	Validations
L1	Data imbalance issue	S1	A K-means SMOTE technique is used to solve the data imbalance issue	V1: Performance comparison of the models
L2	Misclassification due to cross-pairs	S2	A Tomek links technique is used to identify the cross-pairs and remove them accordingly	V2: Table 3 Removal of cross-pairs
L3	Data leakage during training	S3	A simple stratified methodology is used to divide the data based on key attributes into subgroups for training of the model	V3: Equations (1)–(7)
L4	High FPR	S4	A hybrid model of Bi-GRU and Bi-LSTM is used to classify samples precisely and reduce high FPR	V4: Figure 6a,b AUC and PRC curve
L5	Lack of abstract features	S5	A stochastic feature engineering approach is opted to generate abstract features	V5: Table 5

**Table 2:** Metadata Information of SGCC Dataset.

Description	Value
Administering years of the dataset	2014–2016
Total number of benign consumers	38,756
Total number of fraudulent consumers	3616

**Table 3:** Cross-Pairs Identification and Removal.

Total Samples (Before)	Removal of Cross-Pairs	Remaining Samples
10,500	105	10,395

**Table 4:** Performance mapping of the executed models.

Models	F1 Score	Precision	Recall	Accuracy
Proposed	80.7	80.6%	80.9%	88.7%
Existing [33]	76.3	84.3%	74.7%	83.1%
SVM	75.0	62.5%	84.3%	72.5%
DT	75.7	62.3%	79.5%	76.3%
RF	78.2	64.2%	77.6 %	73.6%

**Table 5:** Performance improvement of the proposed model against stochastic feature engineering.

Models	Without Feature Engineering	With Stochastic Features
Proposed Model	88.7%	95%

**Table 6:** Robustness Performance of Proposed Model against Unseen Theft Attacks.

Models	Accuracy	AUC Score	F1 Score
Proposed Model	88.3%	57.6	54.9
Existing Model	86.9%	54.9	53.6.7

**Table 7:** Computational Complexity Analysis.

Input Batch Size	Execution Time Proposed Model (s)	Execution Time Existing Model (s)
50	218	62
100	165	88
150	159	48
200	159	87
250	166	87
300	152	88

## Data Availability

Not applicable.

## Abbreviations

The following abbreviations are used in this manuscript:

AMI	Advanced Metering Infrastructure
APD-HT	Anomaly Pattern Detection Hypothesis Testing
Bi-GRU	Bi-directional Gated Recurrent Unit
AUC	Area Under the Curve
Bi-LSTM	Bi-directional Long Short-Term Memory
CatBoost	Categorical Boosting
CNN	Convolutional Neural Network
DTKSVM	Decision Tree Combined K-Nearest Neighbor and Support Vector Machine
EBT	Ensemble Bagged Tree
ETD	Electricity Theft Detection
DT	Decision Tree
DR	Detection Rate
DG	Distributed Generation
XGBoost	Extreme Gradient Boosting
Fits	Feed-in Tariffs
FN	False Negative
FP	False Positive
FPR	FP Rate
GBCs	Gradient Boosting Classifiers
LGBoost	Light Gradient Boosting
MIC	Maximum Information Coefficient
ML	Machine Learning
NaN	Not a Number
NAN	Neighborhood Area Network
NTLs	Non-Technical Losses
PV	Photo Voltaic
PRC	Precision Recall Curve
RUSBOOST	Random Under Sampling Boosting
RF	Random Forest
SSEA	Semi-Supervised Auto-Encoder
SGCC	State Grid Corporation of China
SMs	Smart Meters
SSDAE	Stacked Sparse Denoising Auto-Encoder
SCADA	Supervisory Control and Data Acquisition
SVM	Support Vector Machine
TLs	Technical Losses
TN	True Negative
TP	True Positive
UP	Utility Provider
WFI	Weighted Feature Importance
*C*	Sample’s Unique Class
*O*	Observations
*p*	Population of the Samples
*S*	Number of Samples
$S_{t}$	Time-Series Data
*T*	Theft Case
σ	Standard Deviation
μ	Mean
